# Phase separation drives the formation of biomolecular condensates in the immune system

**DOI:** 10.3389/fimmu.2022.986589

**Published:** 2022-11-10

**Authors:** Yuqing Wen, Jian Ma

**Affiliations:** ^1^ NHC Key Laboratory of Carcinogenesis, Hunan Cancer Hospital and The Affiliated Cancer Hospital of Xiangya School of Medicine, Central South University, Changsha, China; ^2^ Cancer Research Institute and School of Basic Medical Science, Central South University, Changsha, China; ^3^ Key Laboratory of Carcinogenesis and Cancer Invasion of the Chinese Ministry of Education, Hunan Key Laboratory of Nonresolving Inflammation and Cancer, Hunan Key Laboratory of Cancer Metabolism, Changsha, China

**Keywords:** Phase separation, biomolecular condensates, innate immunity, inflammatory responses, adaptive immunity

## Abstract

When the external conditions change, such as the temperature or the pressure, the multi-component system sometimes separates into several phases with different components and structures, which is called phase separation. Increasing studies have shown that cells condense related biomolecules into independent compartments in order to carry out orderly and efficient biological reactions with the help of phase separation. Biomolecular condensates formed by phase separation play a significant role in a variety of cellular processes, including the control of signal transduction, the regulation of gene expression, and the stress response. In recent years, many phase separation events have been discovered in the immune response process. In this review, we provided a comprehensive and detailed overview of the role and mechanism of phase separation in the innate and adaptive immune responses, which will help the readers to appreciate the advance and importance of this field.

## 1 Introduction

Complex cellular responses underlie the diversity of life activities in living organisms. A wide variety of cellular activities occur in a relatively dynamic equilibrium microenvironment. The cell compartment is essential for organizing the packed cellular space, in order that complex biochemical reactions occur in a spatially and temporally controlled manner (1). The cell compartment is formed by plentiful membrane-enclosed organelles and membraneless compartments. The classic membrane organelles are compartments surrounded by an enclosing or separating biological membrane. Biological membranes often consist of a phospholipid bilayer with embedded, integral and peripheral proteins used in communication and transportation of chemicals and ions. Membrane-bounded organelles provide the physical separation required for specialized processes occurring in the cell compartments to ensure efficient and orderly life activities (2). For instance, mitochondrion consists of an outer smooth membrane and an inner folded membrane, which provide the main place for the aerobic respiration of the cell. The endoplasmic reticulum and Golgi apparatus are membrane-bounded compartments specialized in protein processing, sorting and trafficking through the cell.

There are also a large number of membraneless organelles in cell compartments that provide independent spaces for cellular reactions. These organelles are highly dynamic complexes composed of proteins, nucleic acids or other molecules. For example, stress granules, germ granules and p bodies in the cytoplasm, as well as nucleolus, nuclear speckles and Cajal bodies in the nucleus. As lacking of the biological membrane separating their internal components from the surrounding medium, the molecular mechanism of the assembly and functions of membraneless organelles remained unidentifiable. Mounting evidence has showed that these membraneless compartments are formed by intracellular phase separation, which are collectively called biomolecular condensates (3). In the past, researchers have often used the term “liquid-liquid phase separation (LLPS)” to describe the phenomenon of intracellular biomolecular condensate formation. As researchers learn more about condensates, Tanja Mittag and Rohit V Pappu think that the nomenclature of liquid-liquid phase separation may create confusions. In the absence of specification, this term can be understood to mean that the coexisting phases are a simple and purely viscous liquid (4). However, biomolecular condensates formed from proteins, RNAs or other molecules are not spherical particles of uniform stickiness. Considering the fact that phase separation can be coupled with various other phase transitions in the complex environment *in vivo*, we use the term “phase separation” more succinctly than “liquid-liquid phase separation”, which can physically range from non-viscous (like water at room temperature) to gel-like. The condensates formed by ordinary liquids usually have a spherical shape when not subjected to the external force. However, in cells, forces are always acting on and deforming condensates. The core contents of the biomolecular condensates are automatically either well-mixed or spatially organized, and switch between different material states, in order to dynamically exchange components with the surrounding cytoplasm or nucleoplasm (5–7). Therefore, compared with classical membrane organelles, biomolecular condensates are more flexible.

Biomolecular condensates formed by phase separation play a significant role in a variety of cellular processes, including the control of signal transduction, the regulation of gene expression, and the stress response (8). Epstein-Barr virus proteins, EBNA2 and EBNALP, which mediate virus and cellular gene transcription, can form liquid-like condensates at the superenhancer sites of *MYC* and *Runx3* (9). As a histone H3F3/H3.3 chaperone, DAXX drives liquid-liquid phase condensation by inducing p62 oligomerization (10), which promotes p62 recruitment and NRF2-mediated stress response. Immune response is a comprehensive manifestation of the physiological functions of various parts of the immune system, including a series of physiological reactions such as antigen presentation, lymphocyte activation, immune molecule formation, and immune effects (11, 12). In recent years, many phase separation events have been discovered in the immune response process. Phase separation was involved in the signal transduction process of T cell receptors and B cell receptors (13, 14). When phosphorylated T cell receptors are triggered, the downstream signaling proteins spontaneously separate into liquid-like clusters centered on the transmembrane protein LAT that promote signaling outputs in T cells (13). In the activation of the B cell receptor signaling pathway, scaffold proteins SLP65, CIN85 and lipid vesicles are separated into biomolecular condensates (13, 14). However, this field (phase separation in immune response) is in its infancy, and more research is needed in the future.

Therefore, the current review mainly summarizes about the biomolecular condensates formed by phase separation in the immune system (see **Table 1**). We introduced the multivalent interaction mechanism that drives the phase separation, followed by analyzing the processes that biomolecular condensates activate or participate in innate and adaptive immune processes and the downstream biological effects. At the end of the review, we discussed the direction of future study on phase separation and several questions.

**Table 1:** Phase separation in the immune responses.

Phase separation events	The core component	Mechanisms of phase separation	Biological effect	References
**Innate immune and inflammatory responses**				
Viral factories	RNA virus : rhabdoviruses, paramyxoviruses, filoviruses, etc.	The intrinsically disordered regions of N protein or P protein.	As the hallmark of infection, affecting interferon pathways and antiviral immune responses.	(15–20)
	DNA virus : herpesviruses, adenoviruses, etc.	EBNA2, EBNALP and other virus proteins.		
Stress granules	Ribosome-free mRNA, RNA-binding proteins, translation initiation factors, and the 40S ribosomal subunit.	mRNAs provide scaffolds for multiple related mRNA-binding proteins.	Blocks viral gene expression and replication and contributes to the antiviral innate immune defense.	(21, 22)
Macroplasma vesicles	Proteins and lipids of the macrophage cell membrane.	The critical temperature of LLPS for lipid membrane of macrophages changed.	Two distinct activations of macrophage entry into M1 and M2 condition.	(23)
cGA-STING signaling pathway	cGAS/DNA concentrate is the center. TREX1 is in the outermost layer of concentrate.	The DNA binding sites of intrinsically disordered N-terminal domain and a C-terminal catalytic domain in cGAS.	Promoting the downstream signaling of the cGAS-STING signaling pathway.	(24)
	STING concentrate aggregates the negative regulator TANK1.	The intrinsic disordered regions and dimerization domains.	Preventing overactivation of immune signaling.	(25)
NF-κB signaling pathway	TAK1 is co-located with the N protein, while IKKβ surrounds the TAK1/N/RNA droplet boundary.	The droplets of the N protein and viral RNA provide a space for recruitment of TAK1 and IKK.	Promoting the activation of NF-κB signaling pathway and inducing the inflammatory responses.	(26, 27)
	The N and P proteins of respiratory syncytial virus (RSV) and the p65 subunit of NF-κB.	The low-affinity interactions with N and/or P.	Inhibiting NF-κB signaling pathway.	(28)
	The epigenetic reader ZMYND8 forms liquid compartments with the p65 subunit of NF-κB.	The low affinity, multivalent interactions mediated by the charged amino acids inside IDR regions of ZMYND8.	Inhibiting the macrophage-dependent inflammatory responses.	(29)
**Adaptive immune responses**				
T cell receptor signaling clusters	The phosphorylated T cell receptor complex, ZAP70 and LAT-GrB2-SOS1 as the core concentrate.	The ligand binding and phosphorylation.	Activating T cells and their downstream cellular biological responses.	(30–32)
	CD28 co-locates with PD1 clusters.	Receptors contain multiple phosphorylable tyrosines.	Inhibiting T cell function.	(33)
B cell receptor signaling clusters	SLP65, CIN85 and lipid vesicles.	The proline-rich motif (PRMs) of SLP65 and the nine SH3 domains of trimer CIN85.	Activating the downstream biological responses in B cells	(34–36)
The LLPS regulators	BRD4, FBN1 or TP53	The DNA binding domain of TP53 has amyloidogenic sequences. BRD4 has a unique C-terminal low-complexity domain.	Regulating the tumor immune microenvironment.	(37)

## 2 The multivalent interactions between biological macromolecules drive phase separation

Phase transition and phase separation were originally concepting in the fields of physics and chemistry. Phase transition is the process by which a substance changes from one phase to another. When the external conditions change, such as the temperature or the pressure, the multi-component system sometimes separates into several phases with different components and structures, which is called phase separation. Like the liquid phase, when the temperature changes, it may separate into two immiscible liquid phases, or into a solid phase and a liquid phase. LLPS refers to the phenomenon in which two mixed liquids are separated from each other (38–40). Phase separation can be coupled with various other phase transitions in the complex environment *in vivo*, which physically range from non-viscous (like water at room temperature) to gel-like. Cells also can form independent compartments through phase separation, blocking irrelevant biomolecules outside, so as to carry out orderly and efficient biological reactions (41, 42). However, intracellular “droplets” are not formed directly from liquids, but rather weakly interacting molecules such as proteins, RNAs, and DNAs gathering together to undergo phase transitions to form independent biomolecular condensates (41, 43). The driving force for the occurrence of intracellular phase separation is mainly derived from the multivalent interactions between biological macromolecules. Molecular structures that generate such multivalent interactions include linear repeat domains in folded proteins, the intrinsic disordered regions (IDRs) of proteins, and their RNA binding domains.

The folded proteins with linear repeat domains are strung together by flexible linkers to generate multivalent proteins. Multivalent interactions drive phase separation to aggregate or segregate specific proteins (8). The Src homology 3 (SH3) domain and its proline-rich motif (PRM) ligand, which were initially discovered in tandem arrays in signaling proteins, are capable of multivalent interactions. The Nephrin, neural Wiskott–Aldrich syndrome protein (N-WASP) and Nck constitute a system of natural three-component interaction *in vitro* (44). It was observed that the tail of Nephrin contains three tyrosine phosphorylation (pY) sites, each of which can bind to the SH2 structure of Nck, and the three SH3 domains on Nck can in turn bind to multiple proline-rich motifs in the N-WASP protein. Since phase separation of the Nephrin–Nck–N-WASP signaling pathway on lipid bilayers increases membrane dwell time of N-WASP and Arp2/3 complex, phase separation promotes the activation of Arp2/3 complexes to increase actin assembly (44, 45). Moreover, the nucleolar protein NPM1 has also been shown to undergo phase separation by the multivalent interactions of the folding domains. NPM1 assembles into pentamers *via* its oligomerizing domain and binds to proteins that contain positively charged Arg-rich linear motifs (R-motifs) *via* its two highly conserved negatively charged acidic tracts, termed A1 (residues 34–39) and A2 (residues 120–130) (46). The P body components DCP2 and EDC3 can spontaneously self-assemble into oil-like droplets (47). The multivalent nature of the postsynaptic density proteins SynGAP and PSD95 is critical for the phase separation to occur (48).

Another specialized protein domain that can generate multivalent interactions to drive phase separation is the IDRs, which display a sequence-intrinsic preference for conformational heterogeneity or disorder. The most important feature of the IDRs is that they can serve as scaffolds for multiple distinctive short linear motifs (8, 49).The IDRs typically have low sequence complexity, and contain single amino acid repeats or repeats of specific protein motifs. Some are enriched in charge or polarity of residues (1). Many condensates contain IDR-containing proteins, such as P bodies, germ granules and some nuclear structures. Experiments show that the Caenorhabditis elegans protein LAF-1, a DDX3 RNA helicase found in P granules, phase separates into P granule-like droplets *via* the electrostatic interactions between acidic and basic tracts *in vitro* (50).The N-terminal domain of germ cell-specific protein (DDX4) contains clusters of 8-10 oppositely charged amino acid residues, which is the IDRs *via* cation-pi interactions between aromatic and basic residues. Human Ddx4 and its isolated disordered N terminus spontaneously self-associate both in cells and *in vitro* into structures that are indistinguishable from the cellular DDX4-organelles, by a mechanism of phase separation arising from electrostatic interactions (51). Moreover, the spindle regulatory protein BuGZ is also contains evolutionarily conserved IDRs, and aggregates into biomolecular condensates by the influence of temperature and hydrophobic aromatic residues (52). In addition to amino acid sidechain interactions, the IDRs of RNA-binding proteins FUS concentrated *via* the interactions involving the polypeptide backbone *in vitro*.

Biomolecular condensates often contain nucleic acid components, especially RNA. Due to the negative charge and flexible structure of RNA, it can provide multivalent sites to facilitate phase separation (53).A study of a membraneless organelle showed that DDX4 condensates can bind or deplete RNA under the control of ATP. RNA molecules function as scaffolds and can differentially partition into condensates (54).The proteins in some biomolecular condensates also contain RNA-binding domains. RNA can interact with proteins to regulate phase separation. The systematic experiments and simulations based on coarse-grained models showed that RNA forms stable scaffolds and recruits protein, but RNA–protein interactions compete with RNA base pairing, which can lead to arrested phase separation. FUS is an RNA-binding protein, and in the related RNP model, it was demonstrated that RNA added to FUS would interact with it multivalently to drive phase separation (55, 56).

In short, specialized domains of proteins, RNAs, and other biomolecules form multivalent interactions when they come close to each other. These multivalent interactions miraculously aggregate biomolecules into condensates in the manner of phase separation, and participate in or regulate stress, inflammation, immunity and many other life activities within the cell.

## 3 Phase separation in innate immune and inflammatory responses

Innate immune signaling is triggered when cell surface or cytosolic pattern recognition receptors (PRRs) recognize exogenous pathogen- and endogenous damage-associated molecular patterns to initiate immune and inflammatory responses. Innate immunity is the body’s first line of defense against pathogen invasion. Phase separation are involved in many processes of the innate immunity. Phase separation mediates the formation of condensates of immunogens, such as viruses and stress, to activate innate immunity. Activation of phagocytic cells (e.g., macrophages) also depends on phase separation. Phase separation participates in the activation and regulation of innate immune signaling pathways, including the cGAS and NF-κB signalings. Moreover, phase separation is also closely related to the inflammatory responses. The involvement of phase separation in innate immunity is described below.

### 3.1 Immunogen-associated liquid-like condensates and the antiviral innate immune responses

#### 3.1.1 Virus factories or viral inclusion bodies

The immunogens of pathogens, collectively referred to as pathogen-associated molecular patterns (PAMPs), are pathogenic structures recognized by innate immunity, such as viral RNAs. Viruses are parasitic in living cells, and are a kind of tiny organisms that can use the nutrients of host cells to autonomously replicate their own DNA, RNA, proteins and other life components. After the virus invades the body, it usually achieves its own effective survival by changing the structure or function of the host cell. During infection, the replication and assembly of many viruses occurs in specialized intracellular compartments. Known as viral factories or viral inclusion bodies, these structures condense viral proteins, nucleic acids, and cytokines, carry the essential steps of viral replication, and protect the viral genome from cellular defenses (57–59). Virus factories can be either membrane-bound or non-membrane compartments. In the case of positive-strand RNA viruses, viral factories are associated with membrane rearrangements from the mitochondria or endoplasmic reticulum, leading to the formation of double-membrane vesicles. While for several negative-stranded RNA, double-stranded RNA and DNA viruses, the virus factory has no membrane, and in some cases has been shown to have liquid-like concentrate properties (59).

Herpesviruses and Adenoviruses are DNA viruses. They form membraneless assemblies that merge as the infection progresses and can fuse together in a liquid-like manner (15, 16), which suggest that the formation of viral inclusion bodies is highly similar to phase separation-driven biomolecule condensates. In addition, Epstein-Barr virus proteins, EBNA2 and EBNALP, which mediate virus and cellular gene transcription, are also DNA viruses. Studies have shown that EBNA2 and EBNALP can be induced to undergo liquid-phase separation *in vitro*, and can form liquid-like condensates at the superenhancer sites of MYC and Runx3 *in vivo* (9). Rotaviruses and reoviruses are typical double stranded RNA (dsRNA) viruses that induce the formation of membrane-less organelles. These organelles, also known as viroplasms, are essential for viral genome transcription and replication. It has shown that the viroplasms of rotavirus and reovirus appear to be spherical and can fuse together, suggesting their liquid properties (60–63). Rhabdoviruses, Paramyxoviruses and Filoviruses belong to the order Mononegavirales (MNV) viruses, and their genome consists of a negative sense, single-stranded RNA molecule. The viral factories of these viruses are spherical during the initial stages of infection, suggesting that they could be liquid-like condensates formed by phase separation (17–19). Influenza A virus is also a kind of negative-strand RNA virus and has also been observed to form inclusion bodies during infection (20). Currently, scientists believe that the viral factories of positive strand RNA viruses are associated with lipid membranes and exist in the form of double-membrane vesicles. However, as a kind of positive strand RNA virus, coronavirus may also form inclusion bodies with liquid properties. The experiments *in vitro* have demonstrated that the SARS-CoV-2 N protein contains disordered regions and phase separates from RNA under the regulation of N protein phosphorylation. More mechanisms for the phase separation of viral inclusion bodies are worthy of further exploration (64–66).

pt?>The virus inclusion bodies formed by phase separation are closely related to the body’s innate immunity. Viral phosphoprotein is not only a key factor in activating the body’s innate immunity, but also an important regulator of the formation of viral inclusion bodies. Viral phosphoproteins play a key role in inhibiting interferon production by blocking phosphorylation of the transcription factor interferon regulatory factor 3 (IRF-3) (67, 68).In addition, RIG-1 and MDA5 are intracellular pattern recognition receptors that recognize cytoplasmic double-stranded RNA. If they recognize viral RNA during viral infection, they will activate type 1 interferon and an inflammatory response against viral infection (69, 70). Studies have shown that EBOV VP35 concentrated in viral inclusion bodies largely counteracts innate immunity. It competes with intracellular pattern recognition receptors for binding to double-stranded RNA and inhibits RIG-I signaling and PKR activation, and impairs the function of IFN regulator-activated kinases IKKϵ and TBK-1 (71–73).

#### 3.1.2 Stress granules

Similar to virus factories, stress granules also can be observed during virus infection. The difference is that the virus factory is a liquid-like concentrate that the virus uses to protect itself and stimulates the host to synthesize it, whereas the stress granules are the defense mechanism formed by the host in the face of viral infection. Because the virus itself does not have a complete translation system for self-replication and reproduction, it relies on the host translation system to express viral proteins. Viruses regulate the translation of the host by hijacking and redirecting ribosomes, translation factors, and RNA-binding proteins to meet their survival needs and reproduce offspring (74, 75). In order to resist mass reproduction after viral invasion, cells have evolved highly specialized stress sensors that can detect viral products and actively inhibit host and viral transformation. Stress response is an important sensing mechanism by which cells respond to environmental changes (75). There is a strong link between cellular stress responses and antiviral innate immune signaling pathways. Stress granules are an essential component of the host stress response and are membrane-free condensates of mRNA and protein that contain ribosome-free mRNA and associated RNA-binding proteins, translation initiation factors, and the 40S ribosomal subunit (76–78).Untranslated mRNAs in stress granules are cross-linked to each other, and these mRNAs provide scaffolds for multiple related mRNA-binding proteins. Multivalent interactions formed between protein-protein, RNA-RNA, and protein-RNA drive the phase separation mechanism to form highly dynamic-like stress granules (22, 50, 79).The formation of stress granules results from a stress response-induced translational block. Since viruses require host translational machinery to synthesize viral proteins, stress granules could block viral gene expression and replication. Although stress granules are assembled in response to translation shutdown, under stressful conditions of viral infection, stress granules can serve as an information platform that modulates the stress response and contributes to the antiviral innate immune defense (21, 75).

### 3.2 Macrophages and phase separation

Macrophages are vital phagocytic cells of the innate immune system and are capable of maintaining tissue homeostasis and regulating inflammatory responses to protect organisms from the risk of infection. What’s more is that macrophages function primarily at the forefront of the immune defense. In order to respond promptly and quickly to various external stimuli and signals, macrophages are extremely sensitive to the surrounding environment. Therefore, the activation state of macrophages can often be adjusted by changing environmental conditions to eliminate or repair pathogenic damage and restore tissue homeostasis (23, 80).The two activation states of macrophages under different stimuli are M1 macrophages and M2 macrophages. M1 macrophages are inflammatory macrophages that respond to inflammatory stimuli with bactericidal activity and tumor-suppressive capacity, and produce high levels of pro-inflammatory cytokines. M2 macrophages have immunomodulatory functions by releasing anti-inflammatory cytokines and reducing the production of pro-inflammatory cytokines, and are mainly involved in tissue remodeling, tumor progression, angiogenesis, and wound healing (23, 81).

Lipid membrane components include a large number of lipids and various types of proteins. Lipid membrane composition is also believed to play a role in signal transduction. The formation of lipid rafts or protein domains is a fundamental regulator of protein interactions. Interestingly, cells were processed *in vitro* to generate macroplasma vesicles whose proteins and lipids underwent phase separation under different stimuli (22). Macroplasma vesicles maintain the diversity of native cell membrane proteins and lipids and can be used to study the properties of lipid membrane (23). The nuclei, mitochondria and endoplasmic reticulum were isolated from RAW 264.7 macrophages in resting and activated states for lipidomic analysis of major membrane lipid classes. It was found that with the activation of macrophages, the lipid membrane composition of its different organelles also changed. Following Kdo2-lipid A (KLA)-induced activation of macrophages, the lipid components of organelles underwent profound remodeling, leading to alterations in most lipid classes/subclasses (82, 83). Over a 24h period of stimulation, intracellular cholesterol levels doubled in macrophages, mainly occurring in cholesteryl esters containing saturated and monounsaturated fatty acyl groups. Significant changes in total phosphatidic acid (PA) and phosphatidyl inositol (PI) in organelles were observed after 8–24 h of KLA stimulation of macrophages. Phosphatidic acid increased several-fold upon stimulation at the 24 h time point. Phosphatidylinositol showed a similar trend towards larger fold increases (82, 83). Among them, the phosphatidylinositol in the nucleus increased the most. At the same time, the activation of macrophages doubled the content of phosphatidyl serine (PS) in the nucleus and mitochondria, while the content of phosphatidylserine in the endoplasmic reticulum decreased. However, except for a slight increase in phosphatidyl ethanolamine (PE) content in mitochondria, the phosphatidylethanolamine components in lipid membranes of other organelles did not change much after macrophage activation (82, 83).

Another study reported that under the regulation of different pro-inflammatory and anti-inflammatory stimuli, the critical point at which macrophage lipid membrane undergoes phase separation changes, leading to two distinct activations of macrophage entry into M1 and M2 condition. IFN-γ, LPS and Kdo 2-Lipid A (KLA) increased the critical temperature of macrophage plasma membrane phase separation, and macrophages were activated to enter the M1-type state. IL-4 reduces the critical temperature of macrophage plasma membrane phase separation, and macrophages enter the M2-type state. Macroplasmic membrane vesicle reconstitution experiments in macrophages revealed that lipids may also be key regulators of phase separation in the plasma membrane, suggesting a correlation between the immune response of macrophages and the lipid composition of their plasma membranes (23).

### 3.3 Phase separation in innate immune signaling pathways

#### 3.3.1 cGAS-STING signaling pathway

The cGAS-STING signaling pathway is an important DNA sensing mechanism in the innate immune system. cGAS contains an intrinsically disordered N-terminal domain and a C-terminal catalytic domain, both of which contain DNA binding sites that bind to double-stranded DNA (dsDNA) in the cytoplasm to catalyze adenosine triphosphate (ATP) and guanosine triphosphate (GTP) synthesizes 2’3’-cGMP-AMP (also known as cyclic guanosine monophosphate adenosine monophosphate, cGAMP). cGAMP then acts as the second messenger to bind and activate stimulator of interferon genes (STING) on the surface of the endoplasmic reticulum (84–86). Upon activation, STING recruits TANK-binding kinase 1 (TBK1), which then phosphorylates interferon regulatory factor 3 (IRF3), leading to the translocation of IRF3 to the nucleus, where it transcriptionally induces type I interferon and inflammatory cytokines to trigger immune responses (86–88).The high density of positively charged residues in the N-terminal domain and the identified DNA-binding sites in the C-terminal domain provide the structural basis for multivalent interactions between cGAS and DNA. The multivalent interaction of cGAS with dsDNA drives the phase separation and promotes the formation of liquid-like cGAS/DNA condensates (24). *In vitro* experimental studies have shown that phase separation can also segregate three-element repair exonuclease 1 (TREX1) into the outermost layer of cGAS/DNA condensates. TREX1 is a dsDNA-degrading enzyme that antagonizes cGAS signaling (25, 89). As a major inhibitor of DNA sensing, TREX1 protects against autoimmune diseases by degrading cytoplasmic dsDNA. In this case, phase separation selectively restricted the entry of TREX1 into the cGAS/DNA concentrate, while maintaining a high concentration of dsDNA in the center of the concentrate to interact with cGAS. Such a mechanism of action effectively inhibits TREX1 function and improves the binding efficiency of cGAS to dsDNA to enhance cGAS activity (24).The formation of cGAS/DNA condensates significantly promoted the downstream signaling of the cGAS-STING signaling pathway. It has been recently reported that binding of STING to cGAMP also triggers the formation of liquid-like condensates localized to the endoplasmic reticulum membrane. Phase separation of STING condensates is mediated by its IDR and dimerization domains. STING concentrate mainly aggregates the negative regulator TANK-binding protein 1 internally in response to the activation of cGAMP and prevents overactivation of immune signaling (25, 89).

#### 3.3.2 NF-κB signaling pathway

COVID-19 is still a global epidemic affecting our health and daily life (90).The severity and mortality of COVID-19 are related to the load of the SARS-COV-2 virus and activation of the immune response in host, particularly inflammation (90). When a patient is infected with the SARS-COV-2, virus first attacks the epithelial cells of the human respiratory tract and replicates extensively in the cells, thereby activating the inflammatory response and leading to the formation of inflammatory cytokine storms (91).The main structural protein of SARS-COV-2 is nucleocapsid (N) protein. Studies have shown that viral RNA binds to the N protein to undergo robust phase separation to form biomolecular condensates (26, 27).TAK1 and IKK are key kinases in NF-κB signaling pathway. The N protein and viral RNA droplets provide a space for recruitment of TAK1 and IKK complexes that maximize the interaction between IKKβ and TAK1. The condensate formed by TAK1/N/RNA recruits and activates downstream molecules, such as IKKβ. TAK1 was completely co-located with the N protein, while IKKβ surrounded the TAK1/N/RNA droplet boundary. Once IKKβ is recruited and activated by TAK1, it drifts in the cytoplasm to form IKK complexes and activate downstream molecules, thereby promoting the activation of NF-κB signaling pathway, resulting in the release of multiple cytokines and inducing inflammatory responses (92).

RelA (p65) and p50 of the NF-κB family act as transcription factors when stimulated by upstream signals and participate in the activation of super-enhancers (SEs), which is important for regulating the proliferation and differentiation of B cells after B cell receptor stimulation (93).Studies have reported that NF-κB is involved in SE activation together with other transcription factors or co-activators (94, 95). The aggregation of multiple transcriptional coactivators and mediators with intrinsically disordered regions (IDRs) promotes the formation of phase separation, which favors efficient gene transcription (96).

Johannes N Wibisana et al. observed the characteristics of SE activity mediated by NF-κB in stimulated B cells by means of fluorescence imaging of RelA, and found that 1,6-hexanediol (an inhibitor of LLPS) and JQ1 (an inhibitor of co-activator protein BRD4) can inhibit the formation of stimulus-dependent NF-κB aggregates. Their study showed that liquid condensates mediated by macromolecular phase separation have a critical role for NF-κB SE in transcriptional regulation of B cells (97).

Phase separation not only participates in the activation of NF-κB signaling pathway, but also inhibits NF-κB signaling pathway by forming biomolecular condensates under viral stimulation. As mentioned earlier, inclusion bodies are membraneless liquid-like condensates formed by the viral proteins in cells. Viruses rely on inclusion bodies to facilitate transcription of their genomes and evade the body’s antiviral immune response (18, 98). After respiratory syncytial virus (RSV) infects host cells, NF-κB subunit p65 is isolated into inclusion bodies by phase separation. p65 isolated in the liquid-like condensate cannot translocate to the nucleus to activate the normal expression of pro-inflammatory cytokine genes and other antiviral genes, leading to inhibition of NF-κB signaling (28). In addition, the epigenetic protein ZMYND8 also forms a fluid compartment with p65 to silence the latent superenhancers and inhibit macrophage-mediated inflammation (28). Superenhancers control the polarization and function of macrophages (99, 100). The fusion of ZMYND8 and p65 liquid condensate is enhanced by signal-induced acetylation of p65. Acetylated p65 directs the redistribution of ZMYND8 to potential superenhancers produced in polarized macrophages, recruiting demethylase LSD1 to silence potential super enhancers and inhibit macrophage-dependent inflammatory responses (29, 101).

### 3.4 Phase separation in other inflammatory responses

**Figure 1:**
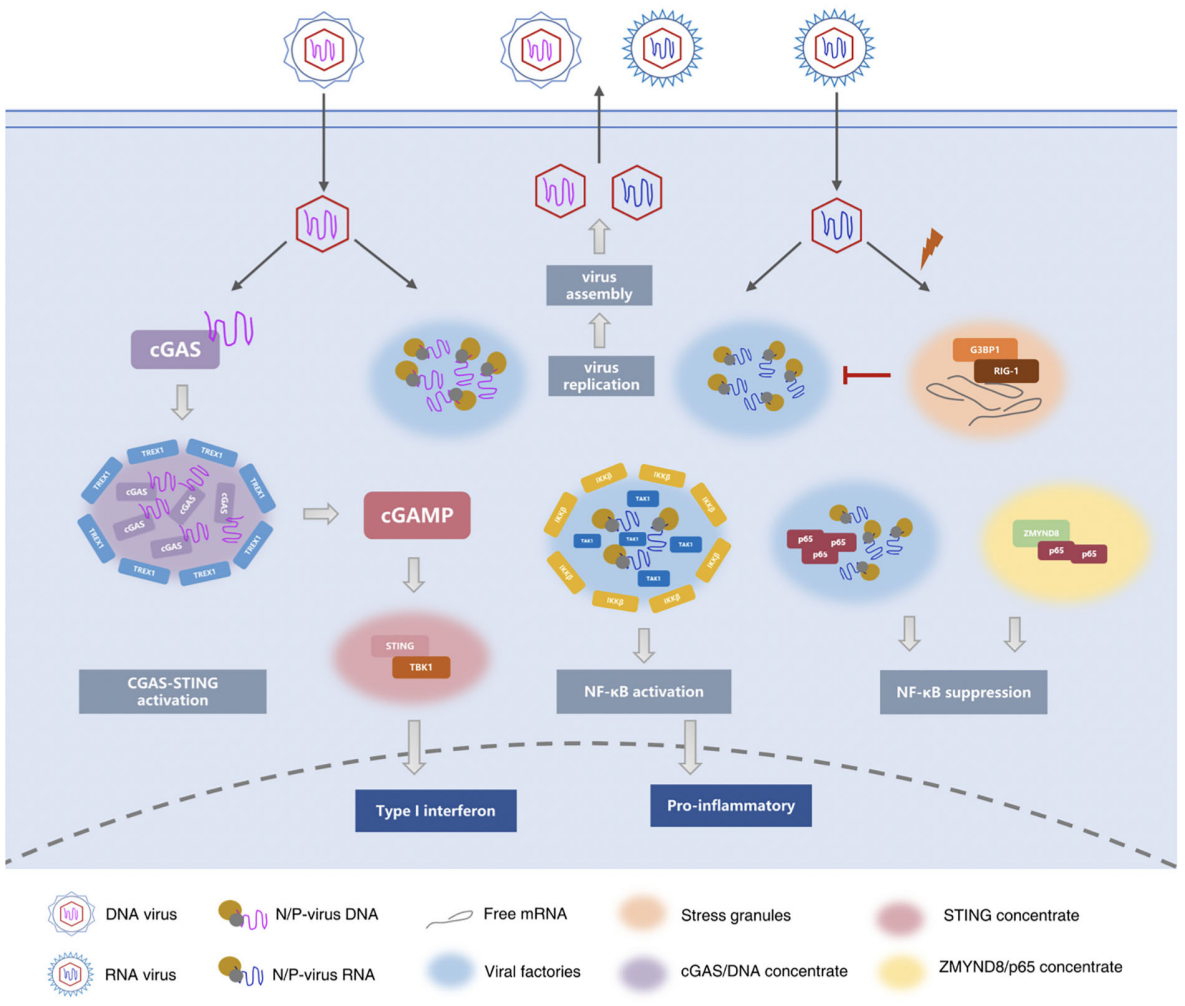
Phase separation in innate immune and inflammatory responses. During infection, the replication and assembly of many viruses occurs in viral factories or viral inclusion bodies. These structures condense viral proteins, nucleic acids, and cytokines, carry the essential steps of viral replication, and protect the viral genome from cellular defenses. (17–19, 58, 59, 63) Similar to virus factories, stress granules (SG) are also be observed during virus infection. The difference is that the virus factory is a liquid-like concentrate that the virus uses to protect itself and stimulates the host to synthesize it, while the stress granules are the defense mechanism formed by the host in the face of viral infection. (74, 75) The cGAS-STING signaling pathway is an important DNA sensing mechanism in the innate immune system. cGAS contains an intrinsically disordered N-terminal domain and a C-terminal catalytic domain, that catalyzes and synthesizes cGAMP. cGAMP then acts as the second messenger to bind and activate STING on the surface of the endoplasmic reticulum. Upon activation, STING recruits TBK1 to promote the transcription of type I IFNs and the expression of inflammatory cytokines to trigger immune responses. (24, 84–88) Viral RNA binds to the N protein of SARS-COV-2 to undergo robust phase separation to form biomolecular condensates. The N protein and viral RNA droplets provide a space for recruitment of TAK1 and IKK complexes. TAK1 was completely co-located with the N protein, while IKKβ surrounded the TAK1/N/RNA droplet boundary. Ultimately, it promotes the activation of NF-κB signaling pathway, resulting in the release of multiple cytokines and inducing inflammatory responses. After respiratory syncytial virus (RSV) infects host cells, NF-κB subunit p65 is isolated into inclusion bodies by phase separation, inhibiting NF-κB signaling pathway. The epigenetic protein ZMYND8 also forms a fluid compartment with p65 to silence the latent superenhancers and inhibit macrophage-mediated inflammation (26–29, 92, 96, 99–101).

p62/SQSTM1 is a multi-domain protein mainly involved in autophagy signal transduction (102, 103). p62/SQSTM1 acts as a hub for multiprotein interactions, integrating signals from multiple pathways, such as selective autophagy, NF-κB, mTOR, and MAPK, which link autophagy to inflammation, immunity, oxidative stress, and many other fundamental biological processes (104–107). p62 forms a flexible filamentous component consisting of N-terminal PB1 domain scaffold and C-terminal binding platform, including folded recognition domain and structurally disordered binding motif. These filaments are part of the cellular p62 body and display the properties of phase separation (108).The cell is a fluid environment, and osmotic pressure balance is essential to maintain the normal volume of the cell and ensure the orderly operation of the cell’s life. The fluid homeostasis of cells is closely related to inflammation. Studies have shown that apoptosis signal-regulated kinase 3 (ASK3) can respond bidirectionally to osmotic pressure to maintain intracellular homeostasis and normal cell volume (109). Under hypertonic stress, ASK3 was kept in liquid-like condensate through phase separation, and osmotic sensitive signal was converted into ASK3 inactivation (110). **Figure 1** summarized the role of phase separation in innate immune and inflammatory responses.

## 4 Phase separation in adaptive immune responses

When the pathogen breaks through the first line of defense formed by the innate immune system, T cells and B cells are activated by antigens carried by antigen-presenting cells. In the adaptive immune response following activation of T cells and B cells, receptors and downstream signal molecules on the surface of immune cells will form condensates supported by phase separation, amplifying the immune cascade signal. Moreover, recent studies have shown that some regulators related to the phase separation can mediate and participate in immune regulation.

### 4.1 Liquid-like condensates and T cell activation

The cell membrane is the basic cellular structure composed of phospholipid bilayer and protein, which not only controls molecular transport, but also regulates cell-cell and cell-environment communication through signal transduction. Cell membranes play a significant role in regulating T cell signaling (111).The fatty acid composition of T cells is closely related to the immune and inflammatory responses (112, 113).Dietary N-3 PUFA has been shown to attenuate the inflammatory responses in a number of clinical studies (114, 115). Recent studies showed that N-3 PUFA may target immune cells and inhibit their activation by changing the dynamic characteristics of the cell membranes. *In vitro* and *in vivo* experiments confirmed that high doses of N-3 PUFA induced the unique phase separation of macroplasma vesicles in T cells, thus changing the fatty acid composition of human CD4+ T cell membrane, followed by increasing membrane fluidity and diverting energy utilization to glycolysis, finally inhibiting the proliferation of CD4+ T cells (116).

The surface receptors and downstream molecular signal transduction of the cell membrane are crucial to activating cellular responses. Clusters of micron and submicron size signals have been reported to be observed on the cell membrane (117, 118). T cell signaling clusters were described as early as 20 years ago (119). Nowadays, T cell receptor (TCR) signaling has been well studied to clarify the effect of such signaling clusters on the signal transduction (118). The key component used to activate T cells during TCR signaling is a transmembrane protein LAT, also known as “linker for activation of T-cell”, which forms biomolecular condensates with growth-factor-receptor-bound protein 2 (Grb2) and son of sevenless homolog 1 (SOS1) by the phase separation (30). When stimulated by antigen, the T-cell receptor complex is phosphorylated by the Src family of membrane-bound protein kinases (Lck). The phosphorylated TCR complex recruits and activates the cytoplasmic tyrosine kinase ZAP70 in its cytoplasmic domain (31, 120),further phosphorylates the linker LAT, and drives the formation of the liquid condensates with LAT-Grb2-SOS1 as the main component. These signal clusters continue to recruit and phosphorylate the adaptive proteins (e.g., Grb2, GADS, and SLP76) to activate the effector proteins at the end of signaling (e.g., SOS1, PLCγ1, Nck, WASP, and Arp2/3) (32). Ultimately the liquid condensates with LAT-Grb2-SOS1 activate T cells and their downstream cellular biological responses, including the aggregation and remodeling of the actin proteins, the influx of calcium, and the activation of the MAPK or the RAS signaling pathway. The Groves group reconstituted the LAT : Grb2:SOS protein condensation on vesicles capable of undergoing liquid-liquid phase transitions. It was observed that the formation of protein condensate can drive the lipid phase transition, which is governed by tyrosine phosphorylation on LAT under isothermal conditions (121). In addition, it has also been reported that the LAT : Grb2:SOS protein condensation occurs immediately downstream of TCR activation, and as a direct result of ZAP70 kinase activation on triggered TCRs (122). SOS plays a key role in Ras activation. The Groves group discovered by a single-molecule assay that the Ras activation is modulated by the LAT : Grb2:SOS protein condensate-driven phase transition (123, 124). Traditionally, Phospholipase Cγ1 (PLCγ1) was considered an enzyme that functions downstream of LAT. PLCγ1 is recruited to LAT microclusters and activated by phosphorylation after TCR activation, resulting in the triggering of downstream calcium and PKC pathways. Recently, it has been reported that PLCγ1 can promote the phase separation of LAT in two ways. On the one hand, PLCγ1 directly cross-links LAT through its two SH2 domains. On the other hand, PLCγ1 can also protect LAT from dephosphorylation by the phosphatase CD45 and promotes LAT-dependent ERK activation and SLP76 phosphorylation (125).Wiscott Aldrich Syndrome protein (WASP) is a functionally powerful protein whose deficiency disrupts cytoskeletal regulation, calcium signaling, and T cell activation. Activation of WASP is critical for the actin filament aggregation. More *in vitro* experiments showed that Nck, WASP and N-WASP act as a clutch that mediates the binding of LAT condensate to actin filaments (126).The linear actin filaments at the distal edge of immunological synapse (IS) assemble into actin arcs of the IS after traversing the outer, Arp2/3-generated region of the IS, which drives the centralization of T cell receptor microclusters (127). Kumari et al. analyzed the structure and dynamics of actin filament networks at immunological synapses of normal and WASP-deficient T cells. They found that the F-actin foci facilitate the later stages of the signaling that activates the T cells, while it cannot be activated when WASP is deficient in cells. This indicates that the LAT condensates or T-cell receptor microclusters regulated by phase separation will play an important role in pathway crosstalk due to the presence of T-cell receptor (TCR) proximal tyrosine kinase cascades (128).

The way in which the phase separation mediates the activation of T cell is not immutable. T cells can be selectively activated to regulate downstream signaling more efficiently. The reconstruction of TCR signaling system *in vitro* revealed that the phase separation among the molecules in the LAT clusters can be mediated by polyvalent interactions. CD45 is a class of negatively charged phosphatases. Because the LAT condensate is also negatively charged, it repels CD45. CD45 was excluded from the phase separation -derived clusters (13). In addition, the selective activation of T cells also regulates the immune response in tumors. CD28 is one of the co-stimulators of T cells and mainly co-locates with PD1 clusters during the activation of T cell. PD1 is normally activated by its ligand PDL1 to recruit phosphatase Shp2 and preferentially dephosphorylates CD28 to inhibit T cell function (33). It indicates that immunotherapy targeting TCR and phase separation can be used in the study of tumor treatment strategies.

### 4.2 Liquid-like condensates and B cell activation

B cells, another important participant in the adaptive immune response, recognize antigens when stimulated by their cell-surface receptors. Downstream effector proteins within the cell are then activated to trigger a signaling cascade leading to B cell activation. Similar to the transmembrane protein LAT that activates T cells, scaffold protein SLP65 is a key component that mediates B cell receptor (BCR) signal transduction, and it can also participate in activating B cells by the phase separation (14, 129). SLP65 is a Src homologous (SH2) domain containing leukocyte protein of 65 kDa, also known as B cell link protein (BLNK) (130, 131). Furthermore, SLP65 has the IDRs containing multiple tyrosine phosphorylation sites and proline-rich motifs (PRMs) that interact with ligands within the SH2 and SH3 domains in specific ways, respectively (34, 132). The IDR is an important mechanism driving phase separation. Recent studies have shown that SLP65, CIN85 and lipid vesicles are separated into condensates to activate B cells (14). SLP65 and CIN85 have polyvalent interactions with the proline-rich motif (PRMs) of SLP65 through the nine SH3 domains of trimer CIN85, and the biophase is separated with the participation of lipid vesicles to form biomolecular condensates (34).SLP65 condensates are performed in the cytoplasm of resting B cells. B cell receptors recruit and activate cytoplasmic splenic tyrosine kinase (Syk) in response to the antigen stimulation. When SLP65 condensates approach the inner surface of the cell membrane, the kinase Syk phosphorylates and activates SLP65. Phosphorylated SLP65 further recruits Bruton tyrosine kinase (BTK) and phospholipase C-γ2 (PLCG2) (35),which in turn trigger downstream biological responses in B cells, such as mobilization of second messenger Ca2+ and nuclear translocation of a key transcription factor NF-κB (36).

### 4.3 Immunomodulation and phase separation

**Figure 2:**
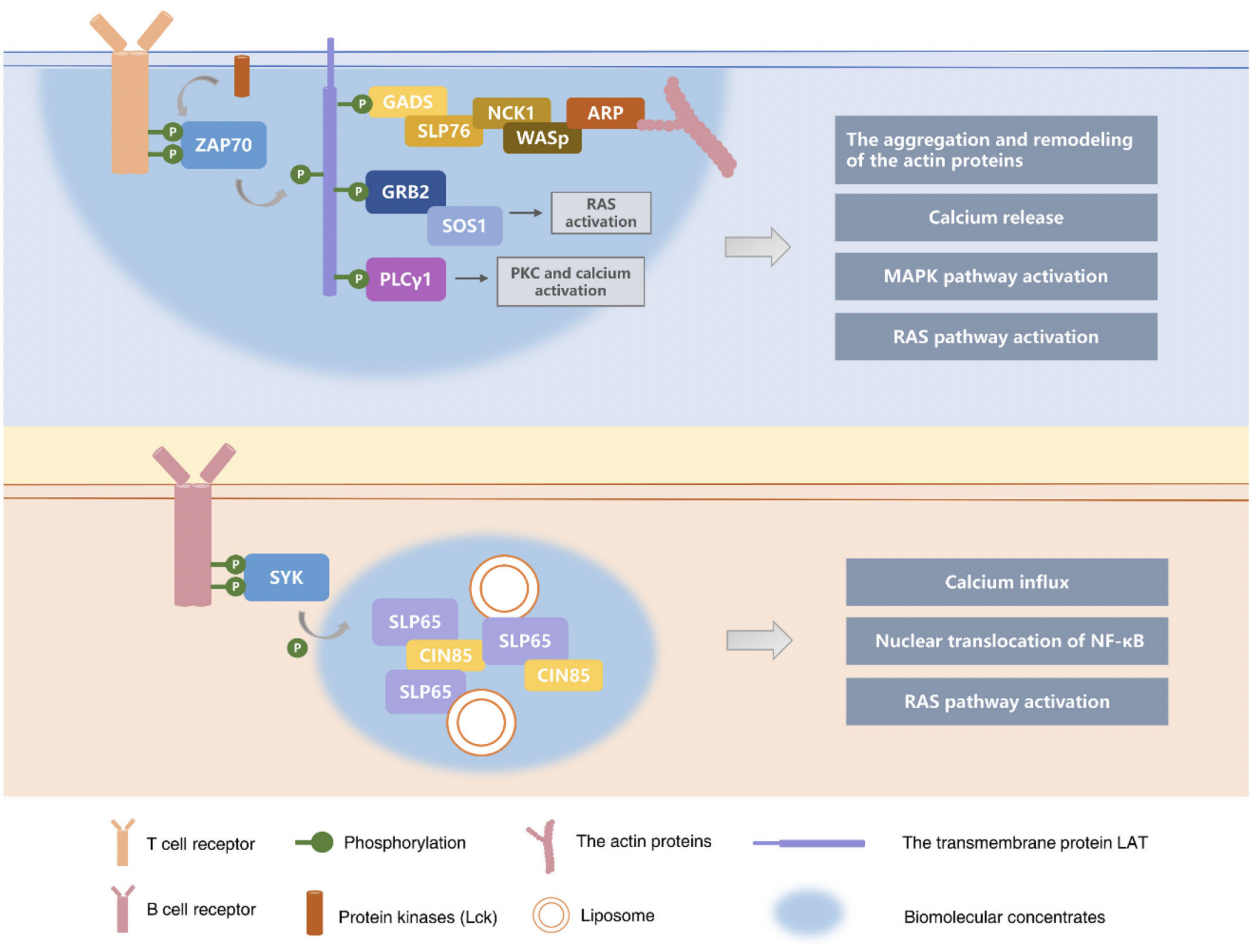
Phase separation in adaptive immune responses. In the adaptive immune response following activation of T cells and B cells, receptors and downstream signal molecules on the surface of immune cells will form condensates supported by the phase separation, amplifying the immune cascade signal. The key component used to activate T cells during TCR signaling is a transmembrane protein LAT (linker), which forms biomolecular condensates with GRB2 and SOS1 by the phase separation. The signal clusters in biomolecular condensates continue to recruit and phosphorylate the adaptive proteins (e.g., GRB2, GADS, and SLP76) to activate the effector proteins at the end of signaling (e.g., SOS1, PLCγ1, Nck, WASp, and ARP2/3). Ultimately the liquid condensates with LAT-GrB2-SOS1 activate T cells and their downstream cellular biological responses. (32, 121–124) Scaffold protein SLP65 is a key component that mediates BCR signal transduction, and it can also participate in activating B cells by phase separation. SLP65 and CIN85 have polyvalent interactions with the proline-rich motif (PRMs) of SLP65 through the nine SH3 domains of trimer CIN85, and the biophase is separated with the participation of lipid vesicles to form biomolecular condensates, which in turn trigger downstream biological responses in B cells, such as mobilization of second messenger Ca^2+^ and nuclear translocation of a key transcription factor NF-κB (34–36, 132).

In recent years, increasing attention has been paid to the role of the phase separation in tumors. It has been reported that the phase separation can also be involved in modulating immune signals in tumors (133). Cancer Genome Atlas (TCGA) data were used to characterize the expression profiles of the phase separation modulators in three gastrointestinal tumor types (e.g., colorectal cancer, gastric adenocarcinoma and esophageal carcinoma). The results showed that the phase separation regulators, such as *BRD4*, *FBN1* and *TP53*, are frequently mutated in all types of digestive tumors (37). BRD4, FBN1 and TP53 have previously been reported to be capable of the phase separation. The study analyzed the association between the phase separation modulators and the infiltration of immune cells in the tumor microenvironment with the help of the bioinformatics. It showed that the phase separation modulators were significantly correlated with the infiltration levels of neutrophils, macrophages, CD4+ T cells, and CD8+ T cells. The phase separation regulates the tumor immune microenvironment by influencing the expression of the phase separation modulators (37). **Figure 2** summarized the role of phase separation in adaptive immune responses.

## 5 Conclusion

Cell compartmentalization effectively guarantees the efficient and orderly progress of various biochemical reactions in cells. Compared with classical membrane organelles, membraneless organelles provide a large number of platforms for intracellular biochemical reactions in a more dynamic and flexible manner. Phase separation is an important mechanism for the formation of membraneless organelles. Phase separation is involved in the immune response process from pathogen invasion to membrane receptor signaling in T cells, and then participates in downstream biological effects such as transcription, calcium ion signaling, and activation of MAPK and RAS signaling pathways. After some viruses invade the body, they stimulate host cells to form viral inclusion bodies under the action of phase separation for their own replication and reproduction. Viral inclusion bodies are also markers of infection. Under the stressful conditions of viral infection, host cells evolve stress granules as information platforms to regulate stress responses and assist the antiviral innate immune defenses. Stress granules are also membraneless organelles formed by phase separation. In the innate immune response, phagocytic cells not only initially clean up and eliminate pathogens, but also play a role in antigen presentation. Macrophages are important phagocytic cells. Under the regulation of different pro-inflammatory or anti-inflammatory stimuli, the critical point at which the lipid membrane of macrophages undergoes phase separation changes, resulting in macrophages entering two distinct activation states, M1 and M2. Furthermore, biomolecule condensates formed by phase separation also regulate important signaling pathways in innate immune responses, such as cGAS and NF-κB signaling pathways. In the adaptive immune response, phase separation is mainly involved in the signal transduction of receptor molecules on the membrane surface of T cells and B cells. In T cells, phase separation drives the formation of liquid-like condensates with LAT-GRB2-SOS1 as the main component. In B cells, SLP65, CIN85 and lipid vesicles are also subjected to phase separation to form liquid-like condensates. Moreover, some studies propose that phase separation can also play a regulatory role in the tumor immune microenvironment by affecting the expression characteristics of phase separation regulators. However, this statement still lacks experimental results to further verify.

Summarizing the research in recent years, the involvement of phase separation in immune response has gradually become a field of great interest to scientists. In addition to what is discussed in this review, the DNA damage response (DDR) plays essential roles to preserve genome integrity. DNA damage can trigger innate immune responses through the accumulation of nuclear DNA in the cytoplasm and the chronic DNA damage response signalling activation (134, 135). Gábor M. Harami et al. found that bacterial single-stranded DNA-binding proteins (SSB) form phase-separated condensates in cells through multifaceted interactions involving structural regions of the protein. SSB condensates can store a variety of DNA repair proteins that specifically interact with SSB (136). Studies also revealed that phase separation may be a critical mechanism for the organization of chromatin structures and the assembly of heterochromatin domains is orchestrated by phase separation (137, 138).Chromatin is generally divided into stretched euchromatin and condensed heterochromatin (139). When active genes move to euchromatin regions, the repressed genes are sequestered in highly condensed heterochromatin regions. It has been reported that early B cell developmental program is accompanied by the repositioning of Ig loci between euchromatin and heterochromatin compartments. Similarly, the loci of T cell receptor reorganize after relocalization and contraction during T-cell development.

Despite some significant progress in this field, there are still many questions about phase separation and immunology that need to be further explored and answered. As we know, the functions of the immune system are immune surveillance, defense and regulation. In addition to immune organs and immune cells, this system also has a large number of immune active substances. In the immune response, immune active substances composed of cytokines, antibodies, and complement play a crucial role in regulation. Could phase separation also drive these actives substances to form liquid-like condensates to control their synthesis or secretion? Although many studies have suggested that phase separation is involved in the regulation of cellular life activities, there is still a lack of research on how we can interfere with the formation of biomolecular condensates by affecting phase separation. The driving force of phase separation is mainly the multivalent interactions formed by the special domains of proteins and nucleic acids. Does this suggest that we can adjust the phase separation to form different condensates by changing the domains of biomolecules? The phase transition in physics refers to the change of a substance from one phase to another under the change of the external environment such as temperature and pressure. Does it mean that we can also regulate the aggregation of biomolecules to form different condensates by changing the internal environmental conditions of cells? The phenomenon of phase separation in immune responses presented here is only a small part of the field (see **Table 1**). With the development of emerging technologies, there are more discoveries waiting for us to explore.

## Author contributions

YW and JM analyzed the literatures and studies, and wrote the manuscript. All authors contributed to the article and approved the submitted version.

## Funding

This work was supported by the National Natural Science Foundation of China (81874170, 82073261), China 111 Project (111-2-12), Natural Science Foundation of Changsha (kq2202125), and Hunan Province Science and Technology Project (2021SK2021).

## Conflict of interest

The authors declare that the research was conducted in the absence of any commercial or financial relationships that could be construed as a potential conflict of interest.

## Publisher’s note

All claims expressed in this article are solely those of the authors and do not necessarily represent those of their affiliated organizations, or those of the publisher, the editors and the reviewers. Any product that may be evaluated in this article, or claim that may be made by its manufacturer, is not guaranteed or endorsed by the publisher.
